# ORAL HEALTH AND DENTAL CARE AMONG OLDER ADULTS IN DIVERSE POPULATIONS: ANALYSES OF LARGE COHORT DATA

**DOI:** 10.1093/geroni/igac059.1096

**Published:** 2022-12-20

**Authors:** Bei Wu, Stephen Shuman, Michele Saunders

## Abstract

Using large cohort surveys, this symposium includes five studies examining the linkages between oral health and systemic conditions, and social and behaviors factors affecting oral health and dental care utilization in older adults. The first study used the English Longitudinal Study of Ageing (ELSA) and analyzed the effect of changes in self-rated dental conditions on memory among adults age 51+. This study also examined the mediation effect of stroke on this relationship. Using the same data (ELSA), the second study examined the longitudinal relationships between cognitive function and changes in diabetes and oral health status. Results showed that participants with co-occurrence of diabetes and poor oral health had an accelerated decline in cognitive function over the study period. The third study was conducted among 8,744 adults age 51+ using data from the Health and Retirement Study over a 10-year period. The authors found that social isolation had a significant effect on dementia onset and that both dental visits and tooth status had a mediating effect on the association. The fourth study analyzed the Medical Expenditure Panel Survey from 2009-2016 and found that individuals with cognitive impairment had a significantly lower probability of annual dental visits. Using data from the Population Study of Chinese Elderly in Chicago collected between 2017-2019, the fifth study found that spousal support was significantly associated with a lower likelihood of having any dental visit. Findings illustrate the importance of understanding how different aspects of social relationships might play a role in dental care use.

